# Intracellular Targeted Nanocapsules Containing Nanobiotherapeutic Suppress Lung, Liver, Breast and Cervix Cancer Cell Lines by Prodrug Activation or Removal of Intracellular Tyrosine

**DOI:** 10.3390/cancers17162698

**Published:** 2025-08-19

**Authors:** ChenHui Zhao, Thomas Ming Swi Chang

**Affiliations:** Artificial Cells and Organs Research Centre, Departments of Physiology, Medicine and Biomedical Engineering, Faculty of Medicine and Health Sciences, McGill University, Montreal, QC H3G1Y6, Canada

**Keywords:** artificial cells, nanocapsules, nanobiotherapeutic, cancers, prodrug, tyrosine, quercetin, o-quinone, metabolic targeting

## Abstract

Background: Many cancer cell lines, such as Hepa 1-6 (liver), A549 (lung), Hela (cervical), and MCF7 (breast), do not overexpress tyrosinase, an enzyme needed to activate the prodrug quercetin into its active form, o-quinone. In addition, these cancers do not rely on extracellular tyrosine for growth, as they can produce small amounts intracellularly. Methods: We investigate two therapeutic strategies using nanocapsules containing polyhemoglobin–tyrosinase (PolyHb–Tyr–nano) for action on (1) the intracellular activation of quercetin to o-quinone and (2) the depletion of intracellular tyrosine. We applied these strategies to the four cell lines listed above. Results: (1) PolyHb–Tyr–nano activates quercetin intracellularly, increasing o-quinone levels and reducing cancer cell viability. (2) PolyHb–Tyr–nano alone suppresses tumor growth by lowering intracellular tyrosine. Furthermore, PolyHb–Tyr–nano shows selective cytotoxicity, with an LD_50_ of 0.7808 mg/mL in Hepa 1-6, compared with an extrapolated LD_50_ of 84,181 mg/mL in the normal liver cells. In contrast, quercetin activation results in an LD_50_ of 2.73 mg/mL in Hepa 1-6 and 74.18 mg/mL in normal hepatocytes. Conclusions: PolyHb–Tyr–nano offers dual therapeutic functions: (1) quercetin prodrug activation and (2) intracellular tyrosine depletion.

## Simple Summary

Unlike melanoma, many cancer cell lines do not overexpress tyrosinase, an enzyme needed to convert quercetin into its active form, o-quinone. In this study, we show that nanocapsules containing polyhemoglobin–tyrosinase (PolyHb-Tyr-nano) can introduce tyrosinase intracellularly to activate quercetin and suppress tumor growth in normal hepatocytes. Quercetin-containing treatments reduce the viability of normal liver cells, whereas PolyHb–Tyr–nano shows no significant cytotoxicity up to the highest dose tested. LD_50_ analysis: In Hepa 1-6, PolyHb–Tyr–nano had the lowest LD_50_ (0.7808 mg/mL), compared with Q-nano (2.7264 mg/mL) and the PolyHb–Tyr–nano + Q-nano combination (1.1648 mg/mL). In normal hepatocytes, no toxicity was observed at the maximum dose (the same dose that eliminated Hepa 1-6); therefore, the LD_50_ was extrapolated and exceeded the tested range (>84,000 mg/mL). Compared with the PolyHb-Tyr-nano, the Q-nano had a lower LD50 in hepatocytes, indicating its potential toxicity in four cell lines: Hepa 1-6 (liver), A549 (lung), Hela (cervical), and MCF7 (breast). We also determined that PolyHb-Tyr-nano alone significantly reduced cancer growth, even without quercetin due to intracellular tyrosine depletion. Cancer cells grow rapidly and require high levels of tyrosine, while normal cells can adapt by synthesizing small amounts. Removing intracellular tyrosine disrupts this balance and selectively suppresses tumor cells. This shows PolyHb-Tyr-nano is an effective antitumor therapy with minimal adverse effects to normal cells compared with a quercetin-related approach.

## 1. Introduction

**Figure 1:**
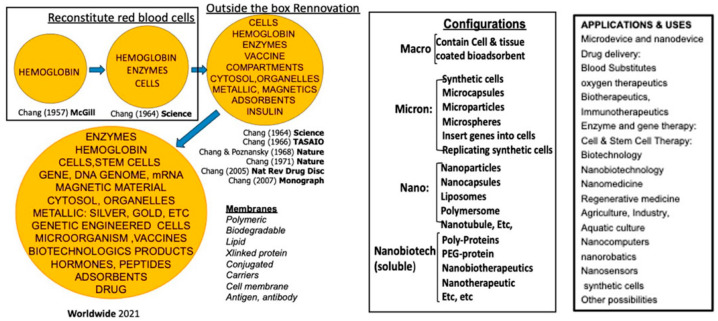
The history of artificial cells including worldwide developments in configurations and applications. From [2] with copyright permission from Francis & Taylor Publisher.

In 1964, Chang [1] first introduced the idea of “artificial cells” in the form of an ultrathin polymer membrane encapsulating biological components such as cells, hemoglobin, or enzymes; over time, this approach has been developed by many laboratories around the word. It has evolved into a variety of configurations and biomedical applications, ranging from micro-and nanoscale capsules to nanoparticles, liposomes, and nanobiotherapeutics [Figure 1] [2].

Herein, we focus on artificial cells’ use in cancer therapy. Tumors have high amino-acid demands to sustain their rapid growth and biosynthesis. An early example of the use of artificial cells was Chang’s use of microencapsulated asparaginase to deplete asparagine and suppress lymphosarcoma in a mice model [3,4]. Meadow’s group used dietary tyrosine restriction for melanoma that showed promise in an animal study but caused weight loss and malnutrition in early clinical attempts [5,6]. Chang’s group introduced a new approach by using polyhemoglobin–tyrosinase (PolyHb-Tyr). This lowered tyrosine in the tumor microenvironment and delayed but did not inhibit tumor growth in mice [7]. Their subsequent use of nanoencapsulation PolyHb-Tyr resulted in the inhibition of growth with no adverse effects in an animal study [8,9].

There is growing interest in targeting tumor metabolism by limiting amino-acid availability [10]. Enzymatic drugs have been engineered to remove specific amino acids [11,12], and multiple nutrient-depleting strategies targeting glutamine, asparagine, and arginine have shown antitumor activity [13,14,15]. However, key challenges remain: (1) many non-melanoma cancers have insufficient tyrosinase to activate certain prodrugs; (2) extracellular depletion can be bypassed by intracellular synthesis of amino acids; and (3) achieving efficient intracellular delivery while minimizing off-target effects is difficult for conventional targeting approaches.

Quercetin is a flavonoid prodrug that requires tyrosinase-mediated oxidation to o-quinone, which can induce ROS-mediated oxidative stress, genotoxicity, and p53-dependent apoptosis [16,17,18,19]. Nanoencapsulation of quercetin prolongs and enhances its antitumor activity in melanoma models [20], but many other tumors underexpress tyrosinase and cannot activate the prodrug. To address these problems, we use nanocapsules containing polyhemoglobin–tyrosinase (PolyHb–Tyr–nano) to pursue two complementary intracellular strategies: (1) the delivery of tyrosinase to enable intracellular activation of quercetin and (2) the depletion of intracellular tyrosine as a metabolic therapy.

**Figure 2:**
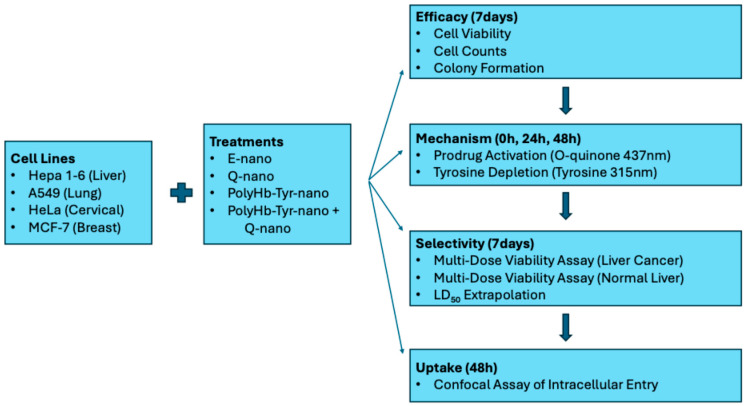
Study schematics. Hepa 1-6, A549, HeLa, and MCF-7 were treated with E-nano, Q-nano, PolyHb–Tyr–nano, or their combination. Efficacy (7 days): cell viability, cell counts, colony formation. Mechanism (0/24/48 h): o-quinone (437 nm) and intracellular tyrosine (PAL, 315 nm). Selectivity (7 days): multi-dose viability in Hepa 1-6 vs. normal hepatocytes; LD_50_ extrapolated in normal hepatocytes. Uptake (48 h): confocal confirmation of intracellular entry.

In the present study, we selected four commonly used cancer cell lines for their global relevance: Hepa 1-6 (liver), A549 (lung), HeLa (cervical), and MCF-7 (breast). Our objectives are to assess the cytotoxic effects and measure the intracellular levels and selectivity relative to normal cells. A schematic of the experimental workflow is provided [Figure 2].

## 2. Materials and Methods

### 2.1. Materials

Purified ultrapure bovine hemoglobin was obtained from Biopure (Cambridge, MA, USA). The Hepa1-6, A549, HeLa, and MCF7 cancer cell lines were purchased from Creative Biogene (Shirley, NY, USA). Glutaraldehyde solution (25%; cat# 00376-500) and poly(D,L-lactic acid) (IV 0.4 dL/g; cat# 16585-10) were obtained from Polysciences Inc. (Warrington, PA, USA). Quercetin (cat# ab120247-100MG) and bisbenzimide H 33342 (cat# ab145597-25MG) were obtained from Abcam Inc. (Cambridge, UK). L-lysine monohydrochloride (≥99%, cat# L8662-1KG), L-tyrosine (≥98% TLC, cat# T2006-1G), and mushroom tyrosinase (EC 1.14.18.1, 3000 units/mg, cat# T3824-50KU) were obtained from Sigma-Aldrich (St. Louis, MO, USA). Phenylalanine ammonia-lyase (PAL; cat# P1016-10UN) and coumarin 6 (≥99%, cat# 546283-100MG) were obtained from MilliporeSigma (Oakville, ON, Canada). CellMask™ Deep Red plasma membrane stain (cat# C10046) was obtained from Life Technologies (Carlsbad, CA, USA). Rat tail collagen I (cat# A1048301) and nutrient agar plates (cat# 50948606) were obtained from Thermo Fisher Scientific (Waltham, MA, USA). Finally, human plateable hepatocytes and uptake-qualified (cat# HMCPUS) and primary hepatocyte maintenance supplements (cat# CM4000) were obtained from Life Technologies.

### 2.2. Cell Culture

The Hepa 1-6 mouse liver cancer line was maintained in Dulbecco’s Modified Eagle’s Medium (DMEM) with 10% fetal bovine serum (FBS). The A549 human lung cancer line was grown in DMEM/F-12 supplemented with 5% heat-inactivated (HI) FBS. The HeLa human cervical cancer line was cultured in DMEM with 10% FBS, and the MCF7 human breast cancer line was maintained in Minimum Essential Medium (MEM) containing 10% HI FBS and 1% non-essential amino acids (NEAA). All cell lines were incubated at 37 °C in a humidified atmosphere with 5% CO_2_ using a HERAcell VIOS 160i CO_2_ incubator (model 2021; serial no. 42621149).

### 2.3. Preparation of PolyHb-Tyr

The reaction mixtures were prepared containing hemoglobin (100 mg/mL) and tyrosinase (6000 U/mL) in 0.1 M potassium phosphate buffer, pH 7.6. In the PolyHb control mixtures, an equivalent volume of buffer replaced the enzyme. Prior to cross-linking, 1.3 M lysine was added at a molar ratio of 7:1 lysine/hemoglobin. The cross-linking reaction was started by the addition of 5% glutaraldehyde at a molar ratio of 16:1 glutaraldehyde/hemoglobin, in four equal aliquots over a 15 min period [7]. The reaction was allowed to proceed for 3.5–48 h at 4 °C under aerobic conditions with constant stirring and was stopped by adding 2.0 M lysine at a molar ratio of 200:1 lysine/hemoglobin. The solutions were dialyzed in physiological saline solution and passed through a sterile 0.45 μM filter. Aliquots (500 μL) of the 16:1 cross-linked preparation were concentrated using 100 kDa micro-concentrators. The samples were centrifuged at 2500× *g* for 55 min at 23 °C, and the retentate was collected. The hemoglobin concentration was determined using cyanmethemoglobin method at 540 nm. PolyHb-Tyr (38.25 mg/mL, 4500 U Tyrosinase) was nanoencapsulated in the aqueous phase with 90 mg of poly-dl-lactic acid (PLA) in the organic phase, following the nanoprecipitation method described previously [9]. The final working concentration for the 1× PolyHb–Tyr–nano solution in the viability studies was 6.4 mg/mL nanocapsules and 225 U of tyrosinase [21].

### 2.4. Preparation of Quercetin Nanocapsules

Quercetin (38.5 mg; MW 302.24 g/mol; Abcam, cat# ab120247-100MG) and poly(D,L-lactic acid) (PLA; 90 mg; Polysciences, cat# 16585-10) were dissolved in acetone–ethanol (3.2 mL:1.6 mL) to form the organic phase. The solution was injected dropwise via a 26 G needle at 3 mL/min into 10 mL of an aqueous phase under magnetic stirring (600 rpm), following the nanoprecipitation method described previously [8]. After addition, the dispersion was stirred for 1 h at room temperature in a fume hood to allow solvent evaporation and nanocapsule formation. Tween 20 (150 µL) was then added to stabilize the suspension. The full physicochemical characterization of the Quercetin and PolyHb–Tyr nanocapsules used in this study has been described in detail in our prior publications [8,20,21]. To provide a concise overview for reproducibility and self-containment, the key properties of the batches employed here are summarized in Table 1.

### 2.5. Measurement of Tyrosinase Activity

Tyrosinase activity was assessed by monitoring the enzymatic conversion of L-tyrosine to dopaquinone, which absorbs at 300 nm. The reaction was carried out in a Perkin Elmer Lambda 4B spectrophotometer, and the absorbance at 300 nm was recorded continuously for 8–14 min. The rate of change in the optical density per minute was calculated and used to determine the enzymatic activity [8].

### 2.6. Three-Dimensional Culture Studies

Cells at ~90% confluency were detached with trypsin, resuspended in DMEM/FBS, and prepared for embedding. In 24-well plates, treatments were set up by adding 71 µL of the drug solution (Q-nano or PolyHb–Tyr–nano) and 35 µL of cell suspension per well, which were then incubated for 20 min to allow excess water to evaporate. For the combination group, 35.5 µL each of Q-nano and PolyHb–Tyr–nano were added to 35 µL of the cell suspension.

On ice, type I collagen mixtures were prepared per well with 355 µL rat-tail collagen I [8], 50 µL 10× PBS, and 8.5 µL 1 N NaOH, to adjust to a neutral pH. The neutral collagen mix was added to each well and gently mixed to combine the cells, drugs, medium, and collagen. The plates were incubated 30 min at 37 °C to allow gelation. The final per-well composition was 106.5 µL drug–cell mixture, 355 µL collagen, 50 µL 10× PBS, and 8.5 µL 1 N NaOH (total ≈ 520 µL).

After gel formation, the culture medium was added gradually over ~1 h; then, the wells were washed 5× to remove the unincorporated drug. The medium was changed daily for 7 days to support growth. On day 7, the gels were digested with 1000 µL collagenase I (4 mg/mL) per well for 30 min at 37 °C with gentle mixing. The cell suspensions were stained with trypan blue, and viable cells were counted using a hemocytometer.

### 2.7. Intracellular Tyrosine Concentration Measurement

Intracellular tyrosine concentrations were measured by first diluting the sample with 1 mL of DMEM/FBS to inactivate the collagenase. The cell suspensions were centrifuged at 16,000× *g* for 20 min at 4 °C to lyse the cells and release the intracellular contents. The supernatant was collected into fresh Eppendorf tubes, which were placed on ice, and the pellet was discarded. Tyrosine was quantified using phenylalanine ammonia-lyase (PAL), which converts tyrosine to trans-coumarate, detectable at 315 nm.

### 2.8. O-Quinone Concentration Measurement

Four treatment groups were tested (*n* = 5): empty nanocapsules (E-nano), free quercetin (Free Q), quercetin nanocapsules (Q-nano), PolyHb–Tyr–nano, and PolyHb–Tyr–nano combined with Q-nano (PolyHb–Tyr–nano + Q-nano). For intracellular o-quinone measurement, 1 mL of cell suspension was collected per sample and centrifuged at 16,000× *g* for 20 min at 4 °C to obtain the lysates. The supernatants were transferred to fresh tubes and kept on ice; the pellets were discarded. Soluble o-quinone was extracted by mixing 100 µL of the supernatant with 1000 µL of methanol, followed by centrifugation at 2000 rpm for 5 min. The methanolic supernatant was used directly for absorbance measurements at 437 nm. Measurements were taken at 0 h, 24 h, and 48 h in 2D cultures.

### 2.9. Colony Study

The cells were spread onto nutrient agar plates and allowed to grow for 24 h. The plates then received the indicated treatments and were incubated at 37 °C, 5% CO_2_. The medium was changed every 2 days until visible colonies formed.

On day 7, plates were gently aspirated and washed 3× with 2 mL PBS. The cells were fixed with 70% ethanol for 15 min, stained with 0.5% crystal violet for 30 min, and washed 3× with PBS. The plates were air-dried and then destained with 10% acetic acid for 15 min. Colonies were counted via light microscopy, and crystal violet was quantified by measuring the absorbance at 590 nm using a UV–Vis spectrophotometer.

### 2.10. Multi-Dose Viability Study

Primary human hepatocytes (Thermo Fisher Scientific (Waltham, MA, USA)) were maintained, following the manufacturer’s protocol, in Williams’ E medium, supplemented with dexamethasone and maintenance supplements. The cancer cell lines were cultured as described above. Three-dimensional collagen cultures were prepared as described in the three-dimensional culture studies subsection. The treatment groups included Q-nano, PolyHb–Tyr–nano, PolyHb–Tyr–nano + Q-nano, E-nano, and PolyHb at 0.25×, 0.5×, 1×, 2×, and 3× (1× defined in Methods: Preparation of PolyHb-Tyr). The treatments were incorporated into the collagen mix for 48 h at 37 °C and 5% CO_2_. After exposure, the drug-containing medium was removed and replaced with fresh medium. The cultures were maintained for 7 days with routine medium changes. On day 7, the gels were digested with collagenase I (4 mg/mL), and the cell viability was determined via trypan blue exclusion using a hemocytometer.

### 2.11. Fluorescence Nanocapsule Entry Study

Coumarin 6–incorporated nanocapsules were prepared by dissolving coumarin 6 in the organic phase with poly-d,l-lactic acid at a dye/PLA ratio of 1:150, followed by the nanoprecipitation method, described earlier, to form PolyHb–Tyr–nanocapsules. The fluorescent nanocapsules were added to the cell cultures and washed to remove excess dye. After incubation, Hoechst dye was applied to stain the cell nuclei [8], and a plasma membrane dye was used to mark the cell borders. The cells were then imaged using an Axio Vert A1 fluorescence microscope. The following filter sets were used: 49 DAPI for Hoechst 33342, 10 Alexa Fluor 488 for Coumarin 6, and 50 Cy5 for AF647. The light source intensity was set to 2%, with an exposure time of 150 ms.

### 2.12. Statistical Analysis

Group differences were assessed using one-way ANOVA followed by Tukey’s HSD post hoc test. Statistical significance was set at *p*-value < 0.05. Significance versus the control is denoted as * *p* < 0.05, ** *p* < 0.01, *** *p* < 0.005.

## 3. Results

### 3.1. Quercetin-Nano, PolyHb–Tyr–nano, and PolyHb–Tyr–nano+ Quercetin-Nano: Effect on Four Cancer Cell Lines

#### 3.1.1. Cell Viability Study

**Figure 3:**
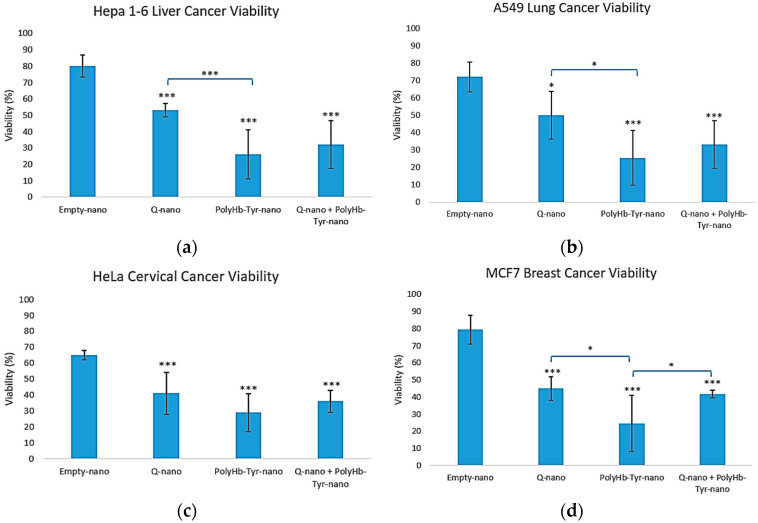
Three-dimensional viability. Cell viability for Hepa 1-6 (**a**), A549 (**b**), HeLa (**c**), and MCF-7 (**d**) after collagen embedding; 48 h exposure to empty nanocapsules (E-nano), quercetin nanocapsules (Q-nano), PolyHb–Tyr–nano, or the combination (PolyHb–Tyr–nano + Q-nano); and a 7 days recovery. Mean ± SD; *n* = 5; one-way ANOVA with Tukey’s HSD; * *p* < 0.05, *** *p* < 0.005 vs. control.

Across all four cancer lines, PolyHb–Tyr–nano caused the loss of viability compared with the control E-nano: 25% in Hepa 1-6 and A549 [Figure 3a,b], 29% in HeLa, and 22% in MCF-7 [Figure 3c,d]. The combination of PolyHb–Tyr–nano + Q-nano produced similar reductions and did not outperform PolyHb–Tyr–nano alone.

#### 3.1.2. Average Live Cell Count

**Figure 4:**
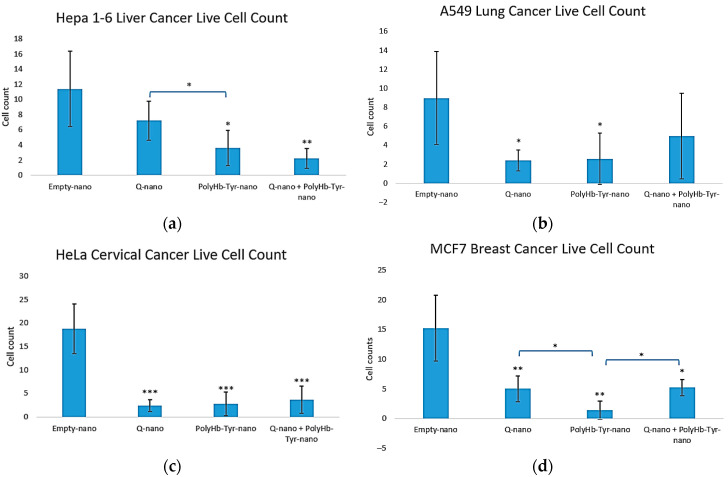
Three-dimensional live cell counts across treatments. Live cell counts for Hepa 1-6 (**a**), A549 (**b**), HeLa (**c**), and MCF-7 (**d**) after collagen embedding; 48 h exposure to E-nano, Q-nano, PolyHb–Tyr–nano, or the combination; and a 7 days recovery. Counts were conducted using trypan blue/hemocytometer. Mean ± SD; *n* = 5; one-way ANOVA with Tukey’s HSD; * *p* < 0.05, ** *p* < 0.01, *** *p* < 0.005.

The live cell counts matched the viability trends. All three treatments reduced the cell numbers versus E-nano across lines [Figure 4a–d]. In Hepa 1-6, PolyHb–Tyr–nano and the combination yielded the fewest live cells [Figure 4a]; in A549 and HeLa, all treatments significantly reduced the counts [Figure 4b,c]; and in MCF-7, PolyHb–Tyr–nano produced the lowest counts [Figure 4d].

#### 3.1.3. Colony Study

**Figure 5:**
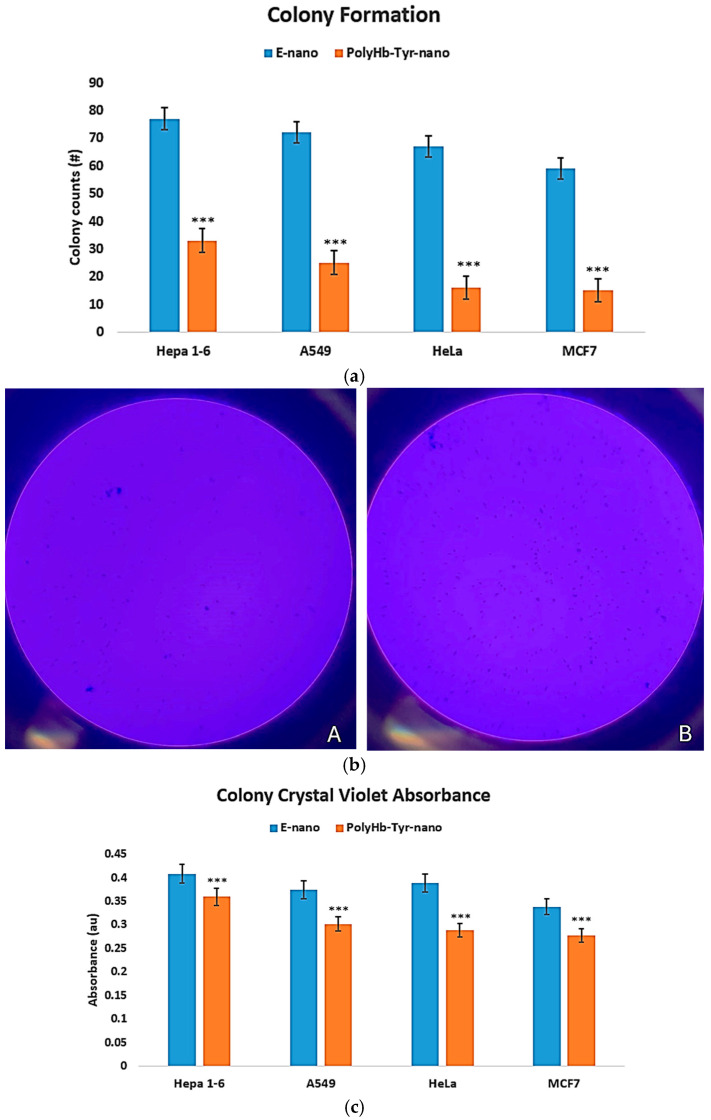
Colony formation assay and crystal violet quantification. (**a**) Colony counts after 7 days on nutrient agar for PolyHb–Tyr–nano versus E-nano in Hepa 1-6, A549, HeLa, and MCF-7. (**b**) Representative plates: Panel (**A**), PolyHb–Tyr–nano; Panel (**B**), E-nano. (**c**) Crystal violet absorbance at 590 nm following acetic acid destaining. Mean ± SD; *n* = 5; *** *p* < 0.005.

PolyHb–Tyr–nano reduced clonogenic survival across all four cancer lines versus E-nano, yielding fewer colonies after 7 days [Figure 5a,b]. The colony size remained unchanged, indicating an effect on the frequency of the surviving clones rather than their growth rate. Crystal violet quantification confirmed lower absorbance in the PolyHb–Tyr–nano group [Figure 5c], consistent with reduced clonogenic outgrowth. This assay was designed to validate the unexpected potency of PolyHb–Tyr–nano seen in the viability tests; therefore, the comparison focused on PolyHb–Tyr–nano versus E-nano.

### 3.2. Quercetin-Nano and PolyHb–Tyr–nano Exert Antitumor Activities by Activating Prodrug to O-Quinone and Reducing Intracellular Tyrosine

#### 3.2.1. Intracellular Tyrosine Measurement Study

**Figure 6:**
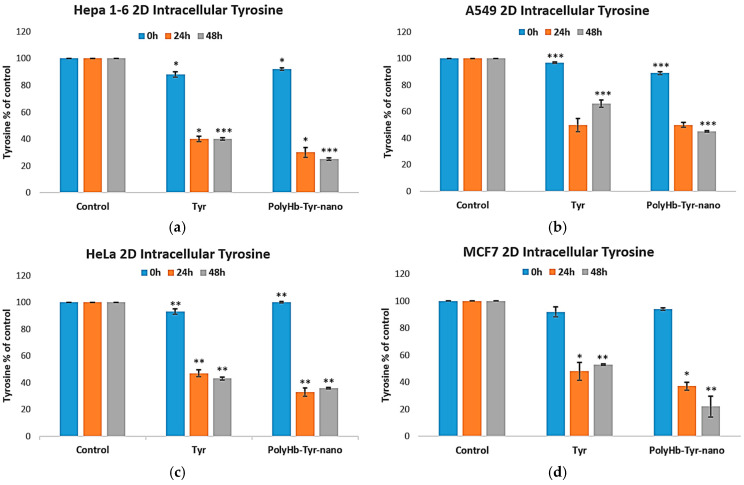
Intracellular tyrosine after treatment. Intracellular tyrosine levels for Hepa 1-6 (**a**), A549 (**b**), HeLa (**c**), and MCF-7 (**d**) measured at 24 h and 48 h in 2D culture. Groups: E-nano (control), free tyrosinase (Tyr), and PolyHb–Tyr–nano. Tyrosine was quantified using the PAL-based readout at 315 nm. Mean ± SD; *n* = 5; one-way ANOVA with Tukey’s HSD; * *p* < 0.05, ** *p* < 0.01, *** *p* < 0.005.

We next tested the tyrosine-depletion mechanism by quantifying the intracellular tyrosine. Compared with the control and free tyrosinase (Tyr) group, PolyHb–Tyr–nano lowered tyrosine at 24 and 48 h across all four lines [Figure 6a–d]. The sustained depletion indicates that nanoencapsulation preserves the tyrosinase activity and enables effective intracellular delivery.

#### 3.2.2. O-Quinone Measurement Study

**Figure 7:**
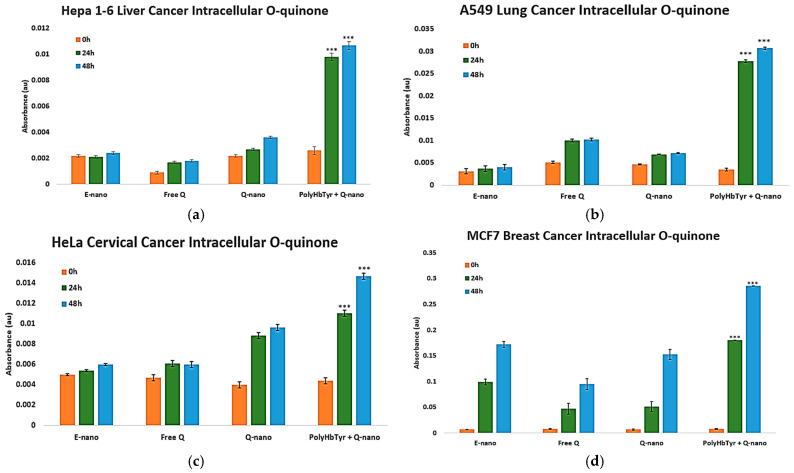
Intracellular o-quinone after treatment. Hepa 1-6 (**a**), A549 (**b**), HeLa (**c**), and MCF-7 (**d**) measured in 2D culture at 0, 24, and 48 h. Cell lysates were prepared using methanol homogenization, and the absorbance was recorded at 437 nm. Groups: E-nano (control), free quercetin (Free Q), Q-nano, and their combination (PolyHb–Tyr–nano + Q-nano). Mean ± SD; *n* = 5; one-way ANOVA with Tukey’s HSD; *** *p* < 0.005.

To test the prodrug activation mechanism, we quantified intracellular o-quinone after treatment with Q-nano or combination therapy. The combination showed a clear time-dependent rise in o-quinone beginning at 24 h and becoming significantly higher by 48 h than the other groups (*p* < 0.0005) across all four lines [Figure 7a–d]. Q-nano alone yielded smaller increases. These kinetics matched the viability trends, supporting the intracellular activation of quercetin as a contributor to the antiproliferative effect.

### 3.3. PolyHb–Tyr–nano Suppresses Liver Cancer Cells Without Cytotoxicity in Normal Liver Cells

**Figure 8:**
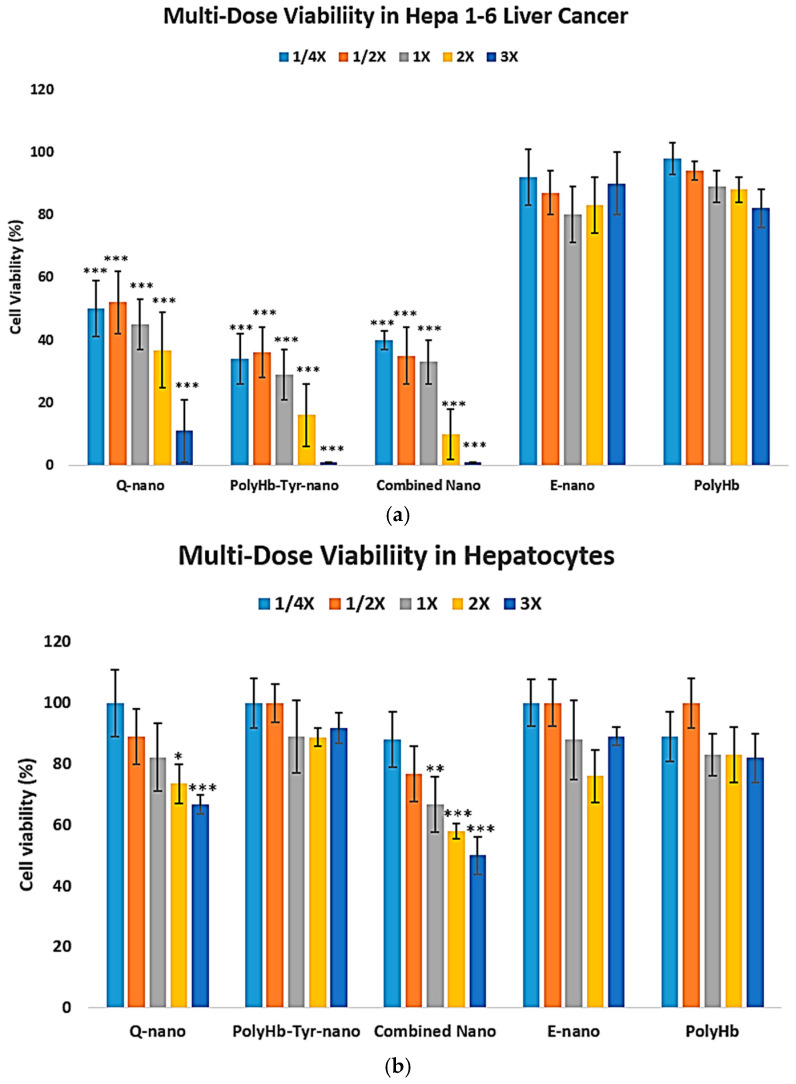
Three-dimensional multi-dose viability: Hepa 1-6 vs. normal hepatocytes. (**a**) Hepa 1-6 viability after collagen embedding, 48 h exposure to E-nano, PolyHb, Q-nano, PolyHb–Tyr–nano, or combination at 1/4×, 1/2×, 1×, 2×, and 3×, followed by a 7 days recovery (**b**). Primary human hepatocyte viability under the same conditions. Viability was assessed using trypan blue/hemocytometer. Mean ± SD; *n* = 5; one-way ANOVA with Tukey’s HSD; * *p* < 0.05, ** *p* < 0.01, *** *p* < 0.005 vs. control at the same dose. 1× nanocapsules concentrations are 6.4 mg/mL with 225 U tyrosinase.

The liver is one of the most sensitive major metabolic organs; therefore, to assess selectivity and safety, we performed a 7-day, 3D collagen, multi-dose study on Hepa 1-6 liver cancer cells [Figure 8a] and primary human hepatocytes [Figure 8b].

In normal hepatocytes, quercetin-containing treatments reduce the viability of normal liver cells, whereas PolyHb–Tyr–nano shows no significant cytotoxicity up to the highest dose tested [Figure 8b]. LD_50_ analysis: In Hepa 1-6, PolyHb–Tyr–nano had the lowest LD_50_ (0.7808 mg/mL) compared with Q-nano (2.7264 mg/mL) and the combination (1.1648 mg/mL) [Table 2]. In normal hepatocytes, no toxicity was observed at the maximum dose (the same dose that eliminated Hepa 1-6) [Figure 8b]; therefore, the LD_50_ was extrapolated and exceeded the tested range (>84,000 mg/mL). Compared with the PolyHb–Tyr–nano, the Q-nano had a lower LD_50_ in hepatocytes, indicating its potential toxicity.

### 3.4. Confirmation of Effective Nanocapsule Cell Entry

**Figure 9:**
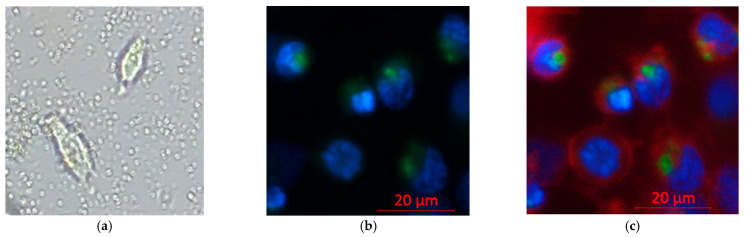
Confocal imaging of nanocapsule uptake in Hepa 1-6 cells. Hoechst 33342 (blue, nuclei), Coumarin 6 (green, nanocapsules), and plasma membrane dye (red, cell borders). Imaging: 20× objective, light source 2%, exposure 150 ms. (**a**) Inverted microscopy of non-stained cells with nanocapsules in the field (**b**). Confocal: green signal within the cytoplasm surrounding blue-stained nuclei (**c**). Confocal with membrane dye: green signal inside the red membrane boundary and in the extracellular space.

To distinguish the intracellular uptake from extracellular accumulation, inverted microscopy showed circular nanocapsules surrounding and co-localizing with cells, suggesting capsule–cell association but not confirming entry [Figure 9a]. Confocal imaging was applied, which confirmed intracellular localization: Coumarin-6-labeled nanocapsules (green) co-localized with Hoechst-stained nuclei (blue) and were not randomly distributed at cell–cell junctions [Figure 9b]. When an additional plasma membrane dye (red) was applied to outline the cell borders, it was evident that both the nucleus and nanocapsules were enclosed within the cell membrane, with no green–blue overlap [Figure 9c]. The signal pattern indicates cytoplasmic and not nuclear localization.

## 4. Discussion

We tested two antitumor strategies: 1. Q-nano with PolyHb–Tyr–nano to activate quercetin to o-quinone and 2. PolyHb–Tyr–nano to deplete intracellular tyrosine. In the 3D collagen culture, PolyHb–Tyr–nano showed the strongest growth suppression across Hepa 1-6, A549, HeLa, and MCF-7, while Q-nano and the combination were active but less potent [Figure 3, Figure 4 and Figure 5]. The corresponding mechanistic assays matched these observations. PolyHb–Tyr–nano lowered intracellular tyrosine at 24–48 h [Figure 6], and the combination increased the intracellular o-quinone over time [Figure 7]. In the intracellular tyrosine depletion assay, only the PolyHb–Tyr–nano group was included. This was an intentional design choice to isolate and directly assess the magnitude of tyrosine depletion without cross-pathway interference from other treatments. The combination group, which introduced quercetin and its o-quinone pathway, could interrupt the specific contribution of PolyHb–Tyr–nano to amino acid depletion. Future studies could include the combination group in this assay to determine whether co-administration influences tyrosinase activity, nanocapsule uptake, or intracellular tyrosine stability. Together, these results support the surprise finding of PolyHb–Tyr–nano’s dominant antitumor mechanism of tyrosine-depletion, rather than prodrug activation.

Selectivity is a key observation. In the 3D multi-dose study, Hepa 1-6 was most sensitive to PolyHb–Tyr–nano, whereas primary human hepatocytes showed no measurable toxicity up to the highest dose tested [Figure 8]. Therefore, the hepatocyte LD_50_ was extrapolated beyond the experimental range [Table 2]. In contrast, the quercetin-related treatments also fit our hypothesis: o-quinone formation can trigger oxidative stress and apoptosis pathways [17,18,19], thus showing potential toxicity and a lower LD_50_ in hepatocytes [Table 2], making PolyHb–Tyr–nano the preferred approach. A simple metabolic explanation is consistent with our data: both normal liver and cancer cells can synthesize tyrosine, but rapidly growing tumor cells have a higher tyrosine demand. When the intracellular tyrosine pool is depleted via PolyHb–Tyr–nano, the cancer cells cannot keep up, while normal hepatocytes compensate via intracellular synthesis and recycling. The intracellular tyrosine decrease supports this view [Figure 6]. Prior in vivo work with PolyHb–Tyr formulations in melanoma also reported good tolerability and no adverse histology in major organs, which supports our hepatocyte results [7,8,9]. After use, the nanocapsule materials are biodegradable: the PLA outer layer hydrolyzes to CO_2_ and H_2_O [22,23], and polyhemoglobin and tyrosinase are broken down to natural components and cleared. Furthermore, modifications with superparamagnetic abilities also showed that an external magnetic field could retain capsules at the injection site, giving more time for intracellular entry, which may further improve local delivery in future studies [21].

In this study, we used 2D and 3D models for different reasons. First, 2D provides robust high-signal readouts for o-quinone and intracellular tyrosine without collagen-related loss; so, it is best for determining the mechanism. Second, 3D collagen better reflects the tumor microenvironment (TME), where the nanocapsules can stay within the TME without rapid clearance. This tests penetration, persistence, and multi-dose responses; so, it is best for determining the efficacy and selectivity. Overall, the results of the 2D mechanisms matched the 3D outcomes [Figure 3, Figure 4 and Figure 5]. However, this is one limitation that we shall address in future experiments. Furthermore, this work focused primarily on the biological evaluation and mechanistic insight of PolyHb–Tyr–nano and combination therapies. Thus, we did not address in vivo tumor models for pharmacokinetics and biodistributions. Further future work will address other gaps, as follows: (1) Expand normal-cell panels and add mitochondrial/ROS/apoptosis markers. (2) Study uptake routes and endosomal escape [24]. (3) Explore more sophisticated models such as organ-on-chips for human-relevant translations. (4) Evaluate the in vivo efficacy, biodistribution, and safety.

In our study, the strong antitumor activity of PolyHb–Tyr–nano in non-tyrosinase-expressing cancers was unexpected. This nanotherapeutic was originally developed for melanoma, where tyrosine depletion is effective, because the tumor depends on external tyrosine for growth [7,8,9]. We initially hypothesized that non-melanoma tumors would not be similarly vulnerable, as their tyrosine metabolism was not considered a major weakness. However, the pronounced inhibitory effect observed in liver, lung, breast, and cervical cancer lines suggests that these tumors may also have elevated tyrosine requirements. Tyrosine sensitivity has been reported by other research groups as a viable antitumor marker in liver, cervical, lung, and breast cancers. Rapidly dividing cancer cells require increased quantities of amino acids to support protein synthesis, energy metabolism, and redox balance. Among these, tyrosine is especially important because it serves as a precursor for proteins, signaling molecules, and key metabolic intermediates. The marked reduction in cancer cell viability we observed following intracellular tyrosine depletion may therefore reflect a broader metabolic vulnerability across different tumor types.

Breast cancer: Metabolomic studies have reported elevated tyrosine levels in breast cancer tissues and patient serum, indicating enhanced tyrosine uptake and metabolism. Tyrosine contributes to ATP production via TCA cycle intermediates and supports biosynthetic demand during tumor progression. Tyrosine kinases, including HER2 and EGFR, are frequently overexpressed in breast cancers, and their activity is closely tied to tyrosine availability [25].

Cervical cancer: Synthetic tyrosine derivatives that cannot function as normal tyrosine inhibit cervical cancer cell growth by competing with endogenous tyrosine for protein incorporation. Cervical cancer cells also overexpress tyrosine phosphatases and present glycoproteins with tyrosine-based motifs, which contribute to oncogenic signaling [26,27,28,29].

Liver cancer: As the primary site of tyrosine metabolism, the liver shows significant alterations in tyrosine handling during hepatocellular carcinoma. HCC is associated with increased circulating tyrosine levels and disrupted tyrosine catabolism. Elevated tyrosine supports proliferation and is linked to the activation of oncogenic pathways such as JAK/STAT and MAPK [30,31].

Lung cancer: Radiolabeled tyrosine tracing studies have shown the preferential accumulation of tyrosine in viable lung tumors, reflecting increased consumption and metabolic activity. Many lung tumors overexpress receptor tyrosine kinases (RTKs), including EGFR, ALK, and MET, which depend on tyrosine-driven signaling cascades [32].

In addition, protein tyrosine phosphatases, tyrosine kinases, and RTKs are established drug targets in oncology [33,34]. Depletion of tyrosine by PolyHb–Tyr–nano could reduce the activity of these enzymes and further disrupt oncogenic signaling. Beyond the four cancers tested in our study, other cancers, such as pancreatic, have also shown elevated tyrosine dependency or dysregulated tyrosine metabolism [35]. This raises the possibility that PolyHb–Tyr–nano therapy could have broader therapeutic applications. The platform could also be enhanced by co-encapsulating enzyme inhibitors that block endogenous tyrosine biosynthesis, such as phenylalanine hydroxylase or tyrosine hydroxylase, to further intensify the tyrosine depletion and potentially improve the antitumor efficacy.

While the exact molecular basis for this sensitivity remains to be determined, it raises the possibility that tyrosine depletion could have broader oncologic applications than previously recognized. Additional mechanistic studies are warranted to explore alternative or complementary pathways that may contribute to the efficacy observed in these tumor types.

## 5. Conclusions

PolyHb–Tyr–nano and its combination with Q–nano act through two anti-cancer mechanisms: intracellular tyrosine depletion and quercetin activation. In 3D models, PolyHb–Tyr–nano showed strong cancer inhibition and lowered the intracellular tyrosine in all lines tested. Most importantly, it remained non-toxic to primary hepatocytes within the tested range, as compared with quercetin-related therapies. While more research is needed to fully understand the potential of PolyHb-Tyr nanocapsules by themselves or in combination with other therapeutics. These results support intracellular metabolic targeting as a viable nanotherapeutic strategy, which encourages expanded mechanistic work, in vivo validation, and eventually clinical translation of this dual-function nanocapsule platform.

## Figures and Tables

**Table 1:** Summary of physicochemical characteristics of Quercetin and PolyHb–Tyr nanocapsules used in this study (compiled from our previously published work [8,20,21]). Both formulations showed comparable properties; values are presented as representative ranges observed across the systems.

Particle Size (nm)	Polydispersity Index (PDI)	Zeta Potential (mV)	Morphology (TEM/SEM)	Encapsulation Efficiency (%)	Preparation Method
180–220	0.12–0.19	−19 to −21	Smooth Spherical	49–70%	Nanoprecipitation

**Table 2:** LD_50_ values from 3D multi-dose viability. The fifty percent lethal dose (LD_50_, mg/mL) for Hepa 1-6 and primary human hepatocytes after treatment with Q-nano, PolyHb–Tyr–nano, and their combination. Values were derived from 7-day 3D collagen cultures (48 h exposure + 7 d recovery; *n* = 5). The hepatocyte LD_50_ for PolyHb–Tyr–nano was extrapolated beyond the tested range, because no toxicity was observed at the highest dose. The 1× concentrations are defined in the Methods.

Treatments	LD50 Dose Liver Cancer (mg/mL)	LD50 Dose Hepatocytes (mg/mL)
Q-nano	2.7264	74.18
PolyHb–Tyr–nano	0.7808	84,181.8
PolyHb–Tyr–nano + Q-nano	1.1648	20.1

## Data Availability

The data supporting the conclusions of this article will be made available upon request.
